# A Comprehensive Review of Recent Trends in Surgical Approaches for Epilepsy Management

**DOI:** 10.7759/cureus.71715

**Published:** 2024-10-17

**Authors:** Tara Sabzvari, Muhammed Aflahe Iqbal, Akash Ranganatha, Jean C Daher, Isabel Freire, Syeda Maham Fatima Shamsi, Oriona Vinishia Paul Anthony, Anusha G Hingorani, Aparita S Sinha, Zahra Nazir

**Affiliations:** 1 Medical School, McMaster University, Hamilton, CAN; 2 General Practice, Muslim Educational Society (MES) Medical College Hospital, Perinthalmanna, IND; 3 General Practice, Naseem Medical Centre, Doha, QAT; 4 Surgery, Jagadguru Jayadeva Murugarajendra (JJM) Medical College, Davangere, IND; 5 Medicine, Lakeland Regional Health, Lakeland, USA; 6 Medicine, Universidad de Ciencias Médicas Andrés Vesalio Guzmán, San Jose, CRI; 7 General Practice, Universidad Central del Ecuador, Quito, ECU; 8 Medical School, Alfaisal University College of Medicine, Riyadh, SAU; 9 Surgery, OO Bogomolets National Medical University, Kyiv, UKR; 10 Medicine and Surgery, Mahatma Gandhi Mission (MGM) Medical College and Hospital, Mumbai, IND; 11 Surgery, Kasturba Medical College, Manipal, Manipal, IND; 12 Internal Medicine, Combined Military Hospital, Quetta, PAK

**Keywords:** adult epilepsy surgery, deep brain stimulation, drug-resistant epilepsy (dre), focused ultrasound (fus), laser interstitial thermal therapy (litt), neuromodulation, responsive neurostimulation, seizure management, surgical outcomes research, vagus nerve stimulation

## Abstract

Epilepsy is a neurological disorder that affects millions of people worldwide, with a significant proportion of patients experiencing drug-resistant epilepsy, where seizures remain uncontrolled despite medical treatment. This review evaluates the latest surgical techniques for managing epilepsy, focusing on their effectiveness, safety, and the ongoing challenges that hinder their broader adoption.

We explored various databases including PubMed, Google Scholar, and Cochrane Library to look for relevant literature using the following keywords: Epilepsy, Resective Surgery, Corpus Collectumy, and Antiepileptic Drugs. A total of 54 relevant articles were found and thoroughly explored.

Recent advancements in surgical interventions include resective procedures such as anterior temporal lobectomy, corpus callosotomy, and hemispherectomy, which have been particularly effective in reducing seizures for specific types of epilepsy. Minimally invasive techniques, including laser interstitial thermal therapy and focused ultrasound, are increasingly being used, offering promising outcomes for certain patient groups. Additionally, neuromodulation methods such as deep brain stimulation, vagus nerve stimulation, and responsive neurostimulation provide alternative treatment options, especially for patients who are not suitable candidates for resective surgery.

Despite these advancements, the full potential of epilepsy surgery is often underutilized due to various challenges. Inconsistent referral practices, a lack of standardized surgical protocols, and significant socioeconomic barriers continue to limit access to these procedures. Addressing these issues through improved referral processes, better education for healthcare providers and patients, and ensuring equitable access to advanced surgical treatments is crucial for optimizing patient outcomes. Future research should focus on overcoming these barriers and assessing long-term outcomes to further enhance the care of patients with epilepsy.

## Introduction and background

Epilepsy, the second most common neurologic disorder in the world, affects approximately 50 million people globally, or 8.5 cases per 1,000 individuals. It affects men and women of all ages and, due to higher risk factors and a greater unmet need for medical care, is more prevalent in low- and middle-income countries [1-3]. The highly variable pathophysiology and variable clinical response to current treatment affect the course of epilepsy treatment, making it daunting to manage in the modern era of medicine.

A seizure is a transient episode of signs and symptoms brought on by abnormally high and synchronized brain neuronal activity. Epilepsy is a neurological disorder that causes an enduring predisposition to generate seizures and the neurobiological, cognitive, psychological, and social consequences that pertain to the condition [4-6]. It is hard to pinpoint a single etiology or pathophysiological pathway responsible for causing epilepsy. Common causes for epilepsy can be roughly divided into genetic causes (chromosomal abnormalities, genetic metabolic disorders, mitochondrial diseases), structural causes (brain tumors/metastases, traumatic brain injury (TBI), hypoxic-ischemic injury, arteriovenous malformations (AVMs)), metabolic causes (porphyrias, inborn errors of metabolism), immune (autoimmune encephalitides) and infectious causes (acute or chronic central nervous system (CNS) infections) [7]. It is, therefore, imperative for physicians to view epilepsy as not a single disease entity but rather a conglomerate of neurological disorders reflecting underlying brain dysfunction [4,5]. The mainstay of treatment of epilepsy traditionally involves the careful use of antiepileptic drugs (AEDs), which result in seizure freedom in about 60%-70% of patients [8]. The most typically used AEDs are levetiracetam, lamotrigine, valproate, or topiramate, with initial therapy often depending on the type of seizure at presentation and side effect profile [9]. Despite this, approximately 30% of patients remain medically refractory even when they are on multiple AEDs. The probability of achieving one year of freedom from seizures after trying two to three AEDs is between 63% and 79%. However, as many as 68% (95% confidence interval (CI) = 65%-70%) of patients with focal epilepsy remain resistant to drug therapy [10,11].

Patients who meet the criteria for drug-resistant epilepsy (DRE) show no response to medical therapy after two trials of tolerated, appropriately chosen, and used AED schedules, either as monotherapy or in combination [12]. Seizures reoccur in approximately 71%-80% of patients who fail two or more AEDs, which is linked to a poor prognosis. There is limited high-quality data available on DRE. Lack of consistency in definitions among experts worldwide limits the ability to obtain robust estimates of the economic and social burden of DRE. However, it is estimated that approximately 80% of healthcare costs surrounding the diagnosis of epilepsy are a direct measure of DRE, making uncontrolled epilepsy not only costly but also associated with adverse cognitive effects, poor quality of life, and increased mortality [11,13,14]. For patients who are diagnosed with DRE, epilepsy surgery has increasingly become a suitable therapeutic option.

Epilepsy surgery is an evidence-based treatment option for people with DRE. The basis for surgical treatment of epilepsy involves the resection of the epileptogenic focus. It strives to control seizures completely without causing further neurologic harm, as well as avoid the long-term complications that are associated with uncontrollable seizures and chronic AED therapy [11,15]. In recent years, surgical approaches for epilepsy management have evolved significantly. Current treatment modalities include surgical resection of the epileptogenic focus (with the aid of MRI and electroencephalography), disconnection procedures (for patients without a resectable focus), minimally invasive ablative procedures (radiofrequency ablation, radiosurgery), and therapeutic devices for seizure control (vagus nerve/deep brain nuclei stimulation) [8,11]. Epilepsy surgery has shown evidence of decreased seizure activity in randomized clinical trials (RCTs) in comparison to giving continued medical therapy [16].

Despite substantial improvements in surgical techniques for treating medically refractory epilepsy, several knowledge gaps remain. Further investigation is required to determine current obstacles to epilepsy surgery. Knowledge-transfer initiatives are required to educate patients and physicians on the relative benefits of epilepsy surgery over antiepileptic medications [11]. Referrals of patients with DRE remain uncommon or, at the very least, far below the expected rate despite data from RCTs and professional practice recommendations supporting the relevance and effectiveness of epilepsy surgery [17]. It is necessary to decide on optimal methods for surgical decision-making protocols and establish patient selection criteria. Further research is also needed regarding the variations in surgical success rates among various populations and healthcare environments. Furthermore, long-term quality-of-life, psychiatric, psychosocial, and cognitive outcomes must be better defined [16].

This literature review aims to overview the most up-to-date advancements in surgical techniques for treating epilepsy. It will review how surgical techniques have evolved, present the most recent data on the effectiveness and safety of these procedures, and outline what still needs to be done in this field to mitigate persistent challenges and move forward. By synthesizing the latest research, this review strives to offer insights into the optimal surgical procedures for epilepsy, ultimately leading to improved patient outcomes and care.

## Review

Epilepsy is defined as a disease in which an individual has two or more unprovoked or reflex seizures greater than 24 hours apart or a single unprovoked or reflex seizure in an individual who has a 60% risk of having another seizure over the next 10 years, or an epilepsy syndrome [18].

Seizures occur when there is abnormal synchronous neuronal firing in a section of the brain or throughout the entirety of the brain when networks are irregularly formed or are perturbed by a structural, infectious, or metabolic disturbance [18]. In children, the most common causes of seizures are genetics, injury due to perinatal insults, and malformations of cortical development [18]. In adults without a genetic predisposition to epilepsy, common etiologies for seizures include encephalitis/meningitis, TBI, and brain tumors [19].

**Figure 1:**
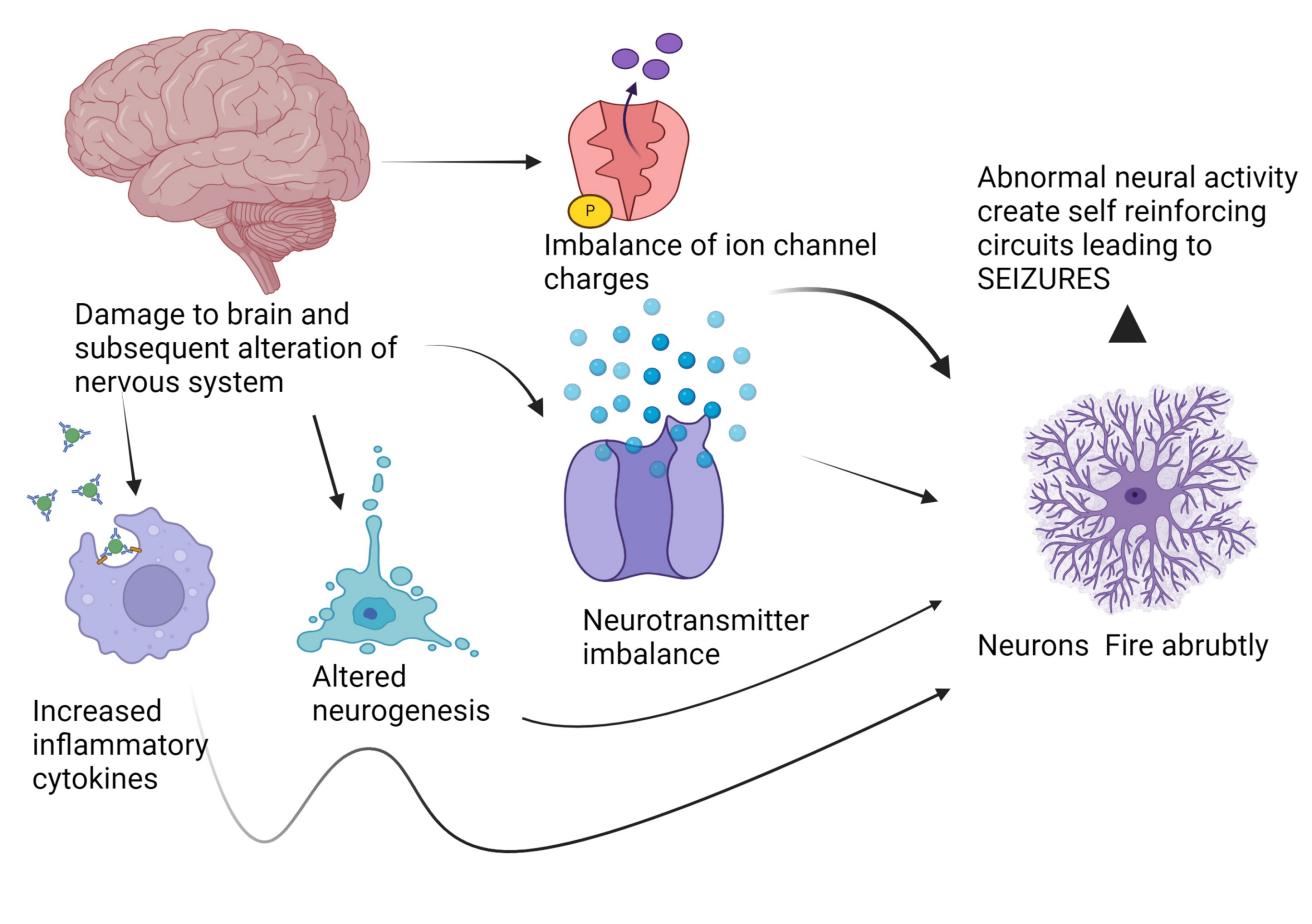
Various inciting factors and their pathophysiology. Source reference: Falco-Walter (2020) [18]. Created by Akash Ranganatha and Zahra Nazir using biorender.com.

In elderly patients, epilepsy is usually the result of primary neurodegenerative disorders, head trauma, and brain tumors. The differences in the etiology of epilepsy among different age groups result in a bimodal prevalence of epilepsy, with genetic/developmental causes peaking in childhood and accumulated injury to the brain (e.g., trauma, tumors) peaking in the elderly [18]. Figure 1 depicts various inciting factors and their pathophysiology in relevance to causing epilepsy [18].

Management

New-Onset Seizures

**Figure 2:**
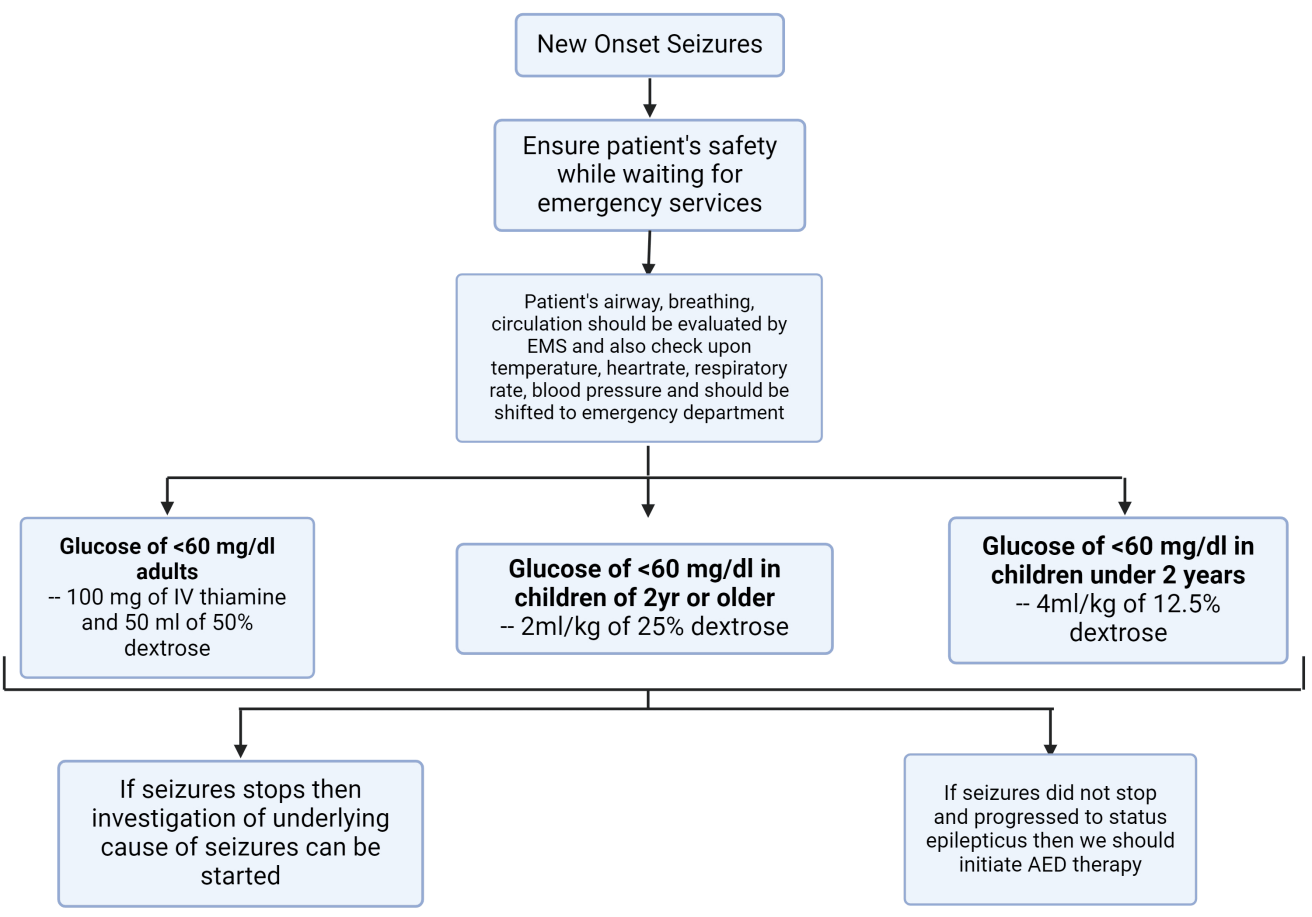
Management of new-onset seizures. Source reference: Bank and Bazil (2019) [21]. Created by Akash Ranganatha using biorender.com.

With any seizing patient, the immediate goal is to ensure the patient’s safety while waiting for the emergency services to arrive [20,21]. Then, the patient’s airway, breathing, and circulation should be evaluated [21]. While being shifted to the emergency department, temperature, heart rate, respiratory rate, and blood pressure should also be checked [21]. Along with this, blood glucose should be monitored. For adults with blood glucose <60 mg/dL, 100 mg of intravenous (IV) thiamine and 50 mg of 50% dextrose should be given. For two-year-old or older children, 2 mL/kg of 25% dextrose, and for children under two years old, 4 mL/kg of 12.5% dextrose should be given [22]. After following all these procedures, if the seizures stop, an investigation for the underlying cause can be started [21]. If the seizures do not stop and progress to status epilepticus, then AED therapy is indicated [21]. Figure 2 sheds light on the management of new-onset seizures [21].

Breakthrough Seizures

Similar to the management of new-onset seizures, initially, it is important to ensure the patient’s safety and confirm that his/her airway, breathing, and circulation are stable [21]. Family members or others who have witnessed previous seizures are often capable of informing whether it is a typical or atypical seizure. Typical seizures are self-limiting and do not require any evaluation by emergency medical services or the emergency department [21]. For atypical seizures (which last for five minutes), rescue medications such as diazepam (rectal) and midazolam (intranasal, buccal) should be administered [22,23]. Rescue medications can be given even after the termination of seizures in patients who have clusters to prevent additional seizures [24]. If the seizures do not rapidly return to baseline mental state, evaluation in the emergency department is appropriate. Laboratory tests such as the basic metabolic panel, complete blood count, and liver function test are done, and a CT scan can be performed for further evaluation [21,25].

Status Epilepticus

Status epilepticus is a seizure lasting five minutes or longer or recurrent seizures activating without returning to baseline in between seizures [26]. Evaluation and monitoring of the airway, breathing, and circulation should be done until reaching the emergency department. During the transit to the emergency department, IV lorazepam 4 mg for adults and 0.1 mg/kg for pediatrics should be given [26,27]. If IV access cannot be obtained, intramuscular (IM) midazolam, 10 mg for adults or children >40 kg and 5 mg for children >13 kg, should be administered [28]. After reaching the emergency department, for the first five minutes, IV lorazepam and IM diazepam, and for the first 20 minutes, IV AEDs (fosphenytoin 20) mg/kg, valproic acid 40 mg/kg, phenobarbital 15 mg/kg) should be given [22]. If the patient continues to have seizures after benzodiazepines and IV AED, then a third line of treatment should be considered [26]. If there are signs of respiratory compromise, the patient should be intubated and treated with a continuous infusion of midazolam, propofol, or phenobarbital [22,26].

Symptomatic Seizures

Symptomatic seizures and neurological diseases mainly include intracranial hemorrhage, subdural hematoma, subarachnoid hemorrhage, brain tumors, and ischemic stroke. For intracranial hemorrhage, subdural hematoma, and subarachnoid hemorrhage, treatment with AED should continue until the underlying insult has resolved. In the case of aneurysmal subarachnoid hemorrhage, craniotomy for aneurysm clipping is performed along with a short course of AED prophylaxis [21]. Brain tumors can also be the reason for precipitating seizures. Seizures can be the presenting complaint of those suffering from brain tumors [21]. Even in this case, AED treatment is indicated (levetiracetam monotherapy 500-700 mg twice a day)[29,30]. Patients with an acute ischemic stroke can also have a new-onset seizure [17]. Recent studies have shown that the administration of tissue plasminogen activator is safe in patients with seizures at the onset of stroke-like symptoms [31]. Similarly, prophylactic treatment with AEDs in post-stroke patients who had no seizures is not recommended [32].

Traditional surgical methods

Epilepsy is a prevalent neurological disorder that impacts more than 70 million people globally. It is marked by a persistent tendency to generate spontaneous seizures, which brings about various neurobiological, cognitive, and psychosocial effects [33-35]. Approximately 30%-35% of people have DRE, which is defined as failure to improve the condition with two adequate trials of AEDs that were appropriate for the patient’s condition [36,37].

Resective surgical procedures have become an integral component of the management of DRE since their introduction in the late 19th century [38]. Currently, resective surgery for drug-resistant focal epilepsies is a well-established treatment option, which is backed by several surgical studies [39,40]. This procedure involves the resection of a specific epileptogenic region, which helps eliminate or reduce the frequency of seizure activity [41]. It may involve resection of the medial structures of the temporal lobe, including the amygdala, hippocampus, and entorhinal cortex, and may also involve resection of the temporal neocortex. An RCT of epilepsy surgery showed that among 80 people with drug-resistant medial temporal lobe epilepsy, 23 of the 40 individuals who underwent anterior temporal lobectomy were free of seizures at their one-year follow-up compared to 3 of 40 individuals who were given medical management alone [42]. A meta-analysis of RCTs and observational studies done in 2015 concluded that resective surgery significantly enhanced the possibility of an individual living seizure-free without impaired awareness by nearly eightfold after one year and increased the likelihood of complete seizure freedom by 15 times [43].

Corpus Callosotomy

The corpus callosum is the bundle of commissural fibers that connects the brain’s two hemispheres and is, therefore, usually responsible for the propagation of epileptic discharge from one hemisphere to another. Thus, severing the corpus callosum can interrupt the transmission of epileptic activity to the contralateral side and help reduce the severity of the seizure. It was first practiced in 1940 by van Wagenen and Herren for the treatment of patients with medically intractable epilepsy [44]. Corpus callosotomy is currently being used successfully for the treatment of drop attacks and epileptic spasms [45-47]. A study by Otsuki et al. showed that 7 out of 10 (70%) patients with epileptic spasms were seizure-free post-corpus callosotomy [48]. Another study done by Baba et al. reported that 43% of patients with epileptic spasms who underwent corpus callosotomy were free of seizures. In another 23% of the patients, a seizure episode was reduced by more than 80%, and a decrease of more than 50% in 13% of the participants. However, no improvement was observed in patients suffering from tonic seizures [49].

In cases where corpus callosotomy is unsuccessful in achieving seizure freedom, additional resection procedures can be performed. A few studies showed promising results, ranging from 43% to up to 71% seizure freedom in patients who underwent resection after corpus callosotomy [46,50].

Hemispherectomy

Hemispherectomy involves the removal of the entire cerebral hemisphere while preserving the basal ganglia. It was first used to treat hemiplegia and intractable epilepsy in 1938 by McKenzie [51]. It was widely used initially for treating the same but was later discontinued mainly due to delayed complications. It was reported that out of the 27 anatomic hemispherectomies performed between 1952 and 1968, 52% led to complications such as hydrocephalus and 33% superficial cerebral hemosiderosis [52]. Several approaches focusing more on disconnection than resection were tried to reduce these complications, finally leading to the development of hemispherectomy.

Hemispherectomy is indicated in patients with intractable epilepsy limited to one cerebral hemisphere. It has a higher success rate in children than adults [53]. Various hemispherectomy techniques have been developed so far, including the peri-insular technique by Villemure and Mascott [54] and the vertical parasagittal technique by Delalande et al. [55]. People undergoing hemispherectomy have a good chance of achieving freedom from seizures, with a success rate ranging from 68% to 94% [56]. In a recent study done in Indonesia, 81.3% of the people who underwent hemispherectomy showed improvement [57].

Minimally invasive surgical techniques for epilepsy

Resective surgery continues to be the primary intervention for refractory epilepsy. However, Dorfer et al. (2020) noted a notable shift toward minimally invasive, neuromodulatory, targeted, disconnective, and ablative procedures. This transition is primarily attributed to recent technological advancements made possible through collaborative efforts among neurologists, neurophysiologists, neurosurgeons, engineers, and scientists [58]. Minimally invasive methods include laser interstitial thermal therapy (LITT), radiofrequency ablation, stereotactic radiosurgery, and, the newest among them, focused ultrasound (FUS) [59]. We explore some of these techniques below.

Laser Interstitial Thermal Therapy

**Figure 3:**
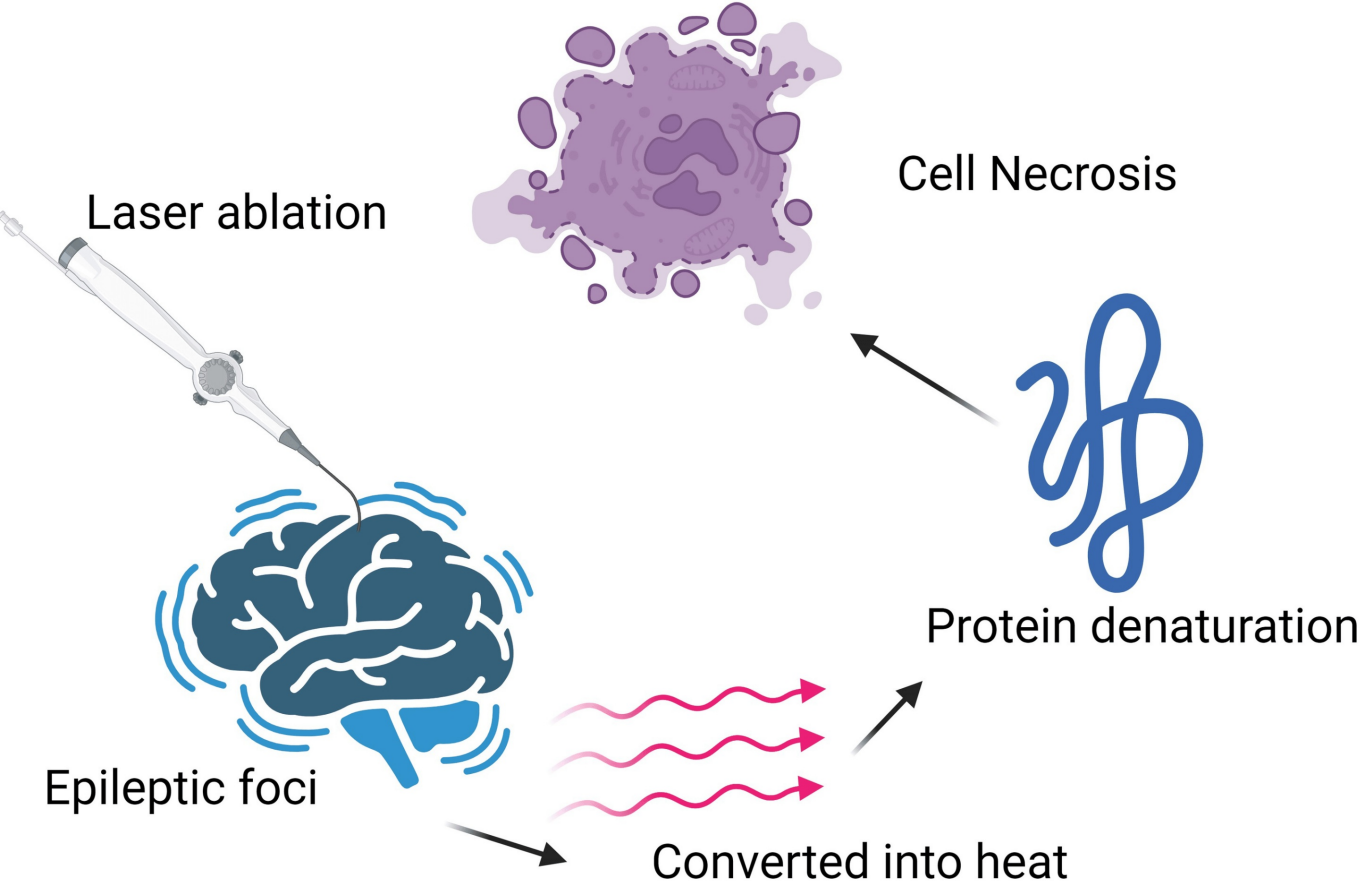
Laser interstitial thermal therapy. Source reference: Patel and Kim (2020) [60]. Created by Zahra Nazir using biorender.com.

DRE, particularly associated with mesial temporal sclerosis (MTS), often necessitates surgical intervention when two AEDs fail to achieve seizure freedom. Traditional surgeries, such as selective amygdalohippocampectomy, carry significant risks. Minimally invasive techniques such as LITT have been developed to enhance safety. LITT uses laser energy to ablate epileptogenic tissue, converting light into thermal energy, leading to protein denaturation and cellular necrosis [60]. Figure 3 demonstrates LITT.

In a study involving 13 patients (nine with MTS), 77% achieved meaningful seizure reduction, with 54% being free of disabling seizures. Of those with preoperative MTS, 67% achieved seizure freedom. No complications were directly attributed to the laser therapy, though one visual field defect occurred due to a procedural error [61].

Emerging evidence indicates that LITT is safe and clinically effective for various neurosurgical conditions, such as low- and high-grade gliomas, brain metastases, radiation necrosis, and seizure foci. However, these findings are predominantly based on small studies (fewer than 50 patients) and retrospective single-institution reports. These studies often exhibit significant variability in quality assurance, complication definitions, and data validation, which limits the generalizability of the results. Additionally, various biases inherent in retrospective institutional studies complicate the interpretation of these datasets [62].

Focused Ultrasound

FUS is an innovative and promising treatment for neuropsychiatric disorders, including epilepsy. FUS employs one or more ultrasound beams at either low or high intensity to modulate brain activity or ablate neuronal tissue. These beams are high-pressure waves generated by a pulse generator and amplified by a transducer. When directed at a focal point in the brain, FUS delivers acoustic energy to the target site [63].

Lee et al. reported that post-treatment MRI scans showed no radiological changes. The cortical lamination appeared normal, and no focal edema was detected in the cerebral white matter. Additionally, low-intensity FUS can be safely delivered to the target area in patients with DRE without significant adverse events [64]. Therefore, FUS has a great advantage over other techniques in terms of safety and adverse effects.

Endoscopic Resection

Endoscopic resection has emerged as a promising treatment for refractory epilepsy associated with hypothalamic hamartomas (HHs) and rare developmental abnormalities of the inferior hypothalamus. A prospective study by Ng et al. investigated 37 eligible patients with refractory epilepsy between 2003 and 2005 who underwent this procedure. Postoperative MRI confirmed the complete removal of HHs from the hypothalamus in 12 patients who had been seizure-free until the last follow-up. At a median follow-up of 21 months (range = 13-28 months), 48.6% of patients were seizure-free, and 70.3% experienced a more than 90% reduction in seizure frequency. It is important to note that three patients did experience permanent short memory loss, while 11 experienced small thalamic infarcts. Despite these outcomes, endoscopic resection patients had a shorter mean postoperative hospital stay than those who underwent transcallosal resection, making it a preferred alternative to traditional operative methods [65].

In a recent study published in the Journal of Neurosurgery (2023), Phillips et al. explored endoscopic approaches for treating mesial temporal lobe epilepsy (mTLE). Conventionally, the surgical approach for mTLE involves a frontotemporal craniotomy followed by an open resection of the anterior and mesial temporal cortex, otherwise referred to as a lobectomy [66]. Although a well-known procedure for neurosurgeons, a major concern of resection has been the associated neuropsychological morbidity, in particular, cognitive deficits and language impairments [67]. The authors of this article evaluated the feasibility of different endoscopic trajectories, for example, endoscopic anterior transaxillary (eATM), endonasal, transorbital, and supra cerebellar transtentorial approaches to identify techniques that directly target the epileptic zone in DRE patients while minimizing interaction with surrounding cortex tissue. Preliminary studies of four patients with mTLE who underwent eATM proved successful without any significant adverse events or deficits, consistently until the one-year follow-up. It is important to note that although eATM has opened the door to less invasive approaches, it is not yet standardized among neurosurgeons as it requires an experienced team of neurosurgeons and otolaryngologists familiar with the techniques, as well as epilepsy experts, along with access to appropriate tools and further knowledge dissemination within the field to explore this surgical innovation within the field of neurosurgery [68-70].

Underutilization of epilepsy surgery: causes and their effects or consequences

Despite its well-documented effectiveness, epilepsy surgery remains underutilized in managing DRE [71-73]. Even with advancements in surgical methods and clear evidence favoring surgical intervention over prolonged medical treatment, many patients who could benefit are not receiving this option [74,75]. This section examines the various barriers that contribute to this underutilization, considering the challenges faced by patients and healthcare providers [55,76]. It also examines the negative impacts of this gap in care. It emphasizes the need for better awareness, improved access, and more inclusive healthcare policies to ensure more patients get the treatment [77-80].

Although surgical management has been identified as a very effective treatment for patients with DRE, it remains underutilized [81,82]. Hence, despite the constant development of new superior strategies and enhanced surgical efficacy, only a limited number of patients who might need this treatment receive surgical intervention [83-85]. Here, we provide insights into partial epilepsy surgery underuse and explore the multifactorial causes and constraints that patients and physicians experience, the existing problems in the healthcare provision, and discrepancies in socioeconomic status [86-94]. It also examines the adverse effects arising from this under-representation, including the effect on epilepsy patients, focusing primarily on the need for improved enhancement and availability of surgical treatments for patients with epilepsy [95-99].

Several studies have proven that epilepsy surgery may help people with DRE reduce or entirely stop having seizures [100]. An RCT observed that 58% of patients with temporal lobe epilepsy achieved a one-year seizure-free status after receiving surgery, while only 8% of the patients in the same group whose condition remained medically managed could achieve this [96]. This shows the superiority of the surgical approach compared to long-term medical treatment [96]. However, despite such convincing arguments, many eligible patients still do not undergo surgery [97]. The following factors explain this situation [98].

One relevant obstacle is the absence of adequate knowledge about epilepsy surgery among patients and clinicians [97]. Some patients need to learn about surgery and its availability as a treatment or gain more accurate perceptions about its risks and benefits [97]. Some of these misconceptions can be compounded when patients and caregivers need to receive adequate information about their conditions from healthcare professionals [98]. Others, including general practitioners, may also have an inadequate awareness of epilepsy surgery indications and their results, which may contribute to patient referral delays to epilepsy centers [98]. Such delays can lead to long, unproductive periods being caught in treatment and poor quality of life among patients [101].

Several advocates also cite systematic barriers within the healthcare domain as a significant factor for epilepsy surgery deficits [99]. These include limited availability of specialized epilepsy centers, long waiting times for surgical assessment, and poor health facilities, especially in the developing world [99]. Consequently, in most parts of the world, skilled neurosurgeons and other professionals are scarce in comprehensive epilepsy care [99]. Further, many clinics may need clear guidelines on when DRE referral should occur and how its management should be handled, implying that these patients receive rather disparate care [100]. These barriers are inherently part of the system, which can pose a challenge to patients who need any surgical operation [102].

Other factors that play a role in contributing to the lack of utilization of epilepsy surgery include patients’ misconceptions about the benefits and risks related to surgery. Patients belonging to a low economic class may sometimes lack proper insurance or may not be economically well-off enough to afford other expenses associated with surgery and postoperative care [101]. Even in countries that boast of social health insurance systems, other related costs such as transport and cost of production, in as much as recurrent losses, are prohibitive [101]. It is also well-recognized that cultural beliefs and stigmatization of this disease can also keep patients from undergoing surgery [102]. Cultural perceptions of epilepsy differ across the globe. They are wholly negative in some places, mainly resulting in social stigmatization, rendering the patient a recluse, and refusal to seek all possible treatment [102].

The risks of not optimizing epilepsy surgery are severe [103]. Patients with DRE who are managed non-operatively are at a higher risk of increased seizure frequency and, therefore, poorer quality of life, cognitive dysfunction, and higher mortality [103]. Seizures may hinder daily tasks, jobs, and social functions, making them expensive in terms of human resources and life [103]. The financial impact of epilepsy that is not well-managed is also significant, comprising increased healthcare costs because of recurrent hospitalizations, emergency department presentations, and chronic pharmacotherapy [104]. These costs burden patients, their families, and the healthcare system [104].

To rectify epilepsy surgery underutilization, greater awareness and patient education, besides physician education, must be emphasized [105]. Awareness and educational programs for the public, physicians, and other healthcare providers, as well as establishing well-equipped epilepsy centers with collaborative care providers, are some ways to go forward [105]. Such efforts may assist in eliminating prejudices, raising awareness, and clarifying the procedure for referral to an evaluation surgery [105]. Furthermore, healthcare policies should focus on removing cost constraints and increasing access to surgical procedures and services, especially in areas that lack adequate surgical capacity [106]. This could entail enhancing the funds allocated to epilepsy treatment, extending insurance coverage, and championing programs that take specialized care to additional localities [106].

Therefore, epilepsy surgery is still underemployed, though available for managing DRE [96]. Given the complexity of the barriers to its use, calls have been made for a collective effort by health professionals, policymakers, and society at large [97]. More patients with DRE can now reach this life-changing intervention through improved awareness, healthcare infrastructure, and lowered socioeconomic disparities [98]. The benefits arising from increasing epilepsy surgery are enormous, as they include better prognosis, reduced overall costs, and improved life expectancy of epilepsy patients [99].

Rehabilitation after epilepsy surgery: the role of neuroplasticity

Epilepsy surgery is considered when a patient cannot be treated with medications and other methods of treating epilepsy. It can significantly change the frequency of the seizures and the patient’s quality of life for the better [107]. However, the ride does not stop here; after-surgery rehabilitation remains essential to enhancing results and entails physiotherapy, occupational therapy, and cognitive training [108]. Rehabilitation is not only about overcoming epilepsy but about working with the power of neuroplasticity, the brain’s impending potentiality to change the pathways it follows, which is crucial for rewiring [109]. As reported by Jacobson et al., after epilepsy surgery, the brain needs to reorganize and regain more typical wiring [110].

Another crucial intervention is cognitive rehabilitation, which helps address cognitive impairments, including those that can arise with epilepsy and its treatment [111]. Approaches such as cognitive behavioral therapy or computer-based training can contribute to the regulation of neuroplastic changes relevant to memory, attention, or executive skills [112]. A critical aspect of physiotherapy is to address problems that may result in motor impairments or hemiparesis after surgery, which can involve encouraging patients to exercise to facilitate mobility [113].

Occupational therapy supports patients in overcoming disabilities, becoming engaged in daily activities, and working through repetitive task practice and functional reorganization [114]. These rehabilitation strategies complement each other and ensure that the overall recovery process addresses physical and cognitive conditions and that patients return to their communities [115].

The role of neuroplasticity in the rehabilitation process after epilepsy surgery is crucial [116]. Research utilizing MRI and other methods demonstrates that rehabilitation therapies promote cortical rearrangement and functional gains [116]. For example, cognitive training has been associated with enhanced activation of the prefrontal cortex and other regions controlling cognitive processes [117]. Further, functional MRI studies have also illustrated enhanced integration and optimization of neural networks in rehabilitated postoperative patients, which underlines the human brain’s plasticity [118].

Specific rehabilitative activities should be chosen according to the location and extent of surgical resection, initial and current cognitive status, and individual goals [119]. It is important to begin rehabilitation early, as the brain is most amenable to change immediately after surgery [120]. Further, it is not rare to need constant follow-up and modification of the rehabilitation program because of patients’ changing requirements and potential for the best long-term benefits [121].

Another significant factor concerns social and psychological support during rehabilitation [122]. This reveals that patients experience various psychological issues after surgery and that including psychological counseling and support groups can improve outcomes and decrease depression and loneliness [122]. Familiarity with the family regarding the condition and its management can also help ensure better support for the patient [123].

Altogether, epilepsy surgery depends not only on the operation performed but also on proper post-surgery rehabilitation that enhances neuroplasticity [124]. This way, patients’ complex needs, which may require cognitive, physical, or occupational therapy approaches, can be addressed effectively, leading to improved rehabilitation outcomes and quality of life [124].

Limitations of epilepsy surgery

Like many medical conditions, a broad spectrum of barriers poses a challenge in delivering a seamless flow of care for patients. There are several considerations, such as macro-scale, the global distribution of access to healthcare, and policies set by governing bodies to micro-scale genetic contributors to disease susceptibility. This list is not exhaustive, as health is multifaceted and as diverse as the humans who acquire it. The main barriers to health include socioeconomic factors, psychosocial considerations, and advancements in technology, cost, and accessibility. Additional considerations can be quantitative, such as morbidity and mortality rates, or qualitative, such as quality of life and systemic racism and discrimination in the healthcare system [125]. Here we analyze the limitations, accessibility, and alternative forms of management within the field of epilepsy surgery.

As evident in recent studies, surgical resection is the medically indicated treatment for medication-resistant epilepsy [126]. However, despite compelling evidence-based endorsements of surgical management, there is considerable variation in referral criteria and policies set by physicians and governing institutions. Common reasons for rejecting surgery include fear of complications, expense, and reservations about benefits. Even though morbidity and mortality from recurrent seizures are much more significant than from surgical treatment, the cost is considerably less than that of a lifetime of disability. The delay in surgical referral has not changed in recent years, and referrals to epilepsy surgery centers have decreased [127].

Patients with DRE, particularly temporal lobe epilepsy, are typically referred for surgical evaluation around 20 years after disease onset in both the United States and Europe. This delay remains unchanged despite significant evidence supporting the benefits of surgical interventions over pharmacologic treatments, as demonstrated by a 2001 RCT and subsequent American Academy of Neurology guidelines [128]. Delayed referrals can exacerbate cognitive and psychosocial impairments, leading to irreversible consequences even if epilepsy control is achieved postoperatively. This delay contributes to an increased risk of premature death associated with refractory epilepsy, though successful surgery can markedly reduce this risk [128].

There is considerable variability in how medically refractory epilepsy is defined among neurologists, which impacts the identification of suitable surgical candidates [129]. This inconsistency extends to the standards for evaluating epilepsy surgery candidacy, with a notable lack of Class 1 evidence supporting extratemporal neocortical resections, corpus callosotomy, and hemispherectomy [130]. Additionally, the absence of standardized measurement tools for disability in refractory focal epilepsy complicates the assessment of treatment effectiveness and patient outcomes [125]. Such variability and lack of standardization hinder the ability to provide consistent and evidence-based care.

In a study of 796 neurologists by Robert et al. in 2014 [131], more than 50% of neurologists who followed patients with epilepsy required patients to be drug-resistant and to have at least one seizure per year before considering surgery. Nearly half failed to define DRE correctly. From these responses, almost 75% agreed that healthcare resources are the most significant barrier to surgery for epilepsy patients. Even within a highly specialized field, there are issues surrounding the diagnosis, treatment, and timing of the treatments given that the patients have access to, including knowledgeable, trained doctors with sufficient patient funds and equipped facilities to deliver these healthcare services. Tying into this is the dissemination of information and patient education by physicians with regards to adverse outcomes and risk of surgery, with some specialists being ill-informed on the prognosis of different management strategies due to lack of up-to-date information and general apprehension, which is, in turn, transmitted to patients by instilling fear for seeking alternative solutions than drug-therapy. This leaves patients hopeless and fearful in attempts to improve their quality of life.

Epilepsy surgery does have risks and can lead to severe complications, with one-third of patients experiencing adverse outcomes [129]. These complications include cognitive deficits, which are common after surgical interventions such as anterior temporal lobectomy, and other issues such as cerebrospinal fluid leaks and infections [126]. Moreover, surgical options often result in minor yet predictable cognitive deficits, though some may experience improvement postoperatively. The risk of these adverse effects, concerns about postoperative recurrence, and the impact on a patient’s quality of life pose a significant limitation to surgical treatment [126]. However, evidence suggests that the benefits of surgery for suitable candidates outweigh the disadvantages posed by surgical risks.

Access to epilepsy surgery is constrained by several factors, including inadequate healthcare resources and variable physician knowledge and referral practices [132]. The lack of consistent and timely referrals results from neurologists’ insufficient understanding of the benefits of surgery and the complexities involved in diagnosing medically refractory epilepsy [128]. The delay in referral and physicians’ reluctance to initiate surgery due to perceived risks or inadequate knowledge significantly limit patients’ access to potentially life-changing interventions [129].

Socioeconomic status, minority status, and educational background contribute to disparities in accessing epilepsy surgery. Patients often hold exaggerated fears about the risks associated with surgery, influenced by misinformation and negative attitudes toward surgical options [133,134]. These misconceptions can result in patients rejecting or delaying surgery despite its potential benefits. Furthermore, stigma and social discrimination toward epilepsy can exacerbate these issues, leading to the underutilization of advanced treatments such as resective brain surgery [131].

In high-income countries, there remains a significant disparity between the availability of state-of-the-art surgical facilities and the actual rate of surgical interventions [134]. The situation is even more dire in resource-poor settings, with limited access to surgical options, long waiting periods, and high costs exacerbating the challenges [135]. These settings’ lack of infrastructure and expertise severely restricts patients’ ability to receive timely and appropriate surgical care.

While AEDs are the primary treatment for epilepsy, they often fail to control seizures in approximately one-third of patients [133]. Alternative treatments include ketogenic diets, vagus nerve stimulation (VNS), and responsive neurostimulation [136]. Surgical resection can be highly effective, with up to 76% of patients achieving seizure freedom [126]. The ketogenic diet and VNS offer non-invasive alternatives for patients unable to undergo surgery, though they come with their own side effects and limitations [126].

The ketogenic diet and other dietary therapies, such as the medium-chain triglyceride diet and the modified Atkins diet, are used as alternative treatments. Despite poor supporting evidence, some claim a 10% seizure-free rate and 60% seizure reduction with diet change [134]. However, their potential benefits are outweighed by associated side effects and require rigorous adherence, which can impact their accessibility [137]. Examples primarily include gastrointestinal symptoms (vomiting, constipation, diarrhea, abdominal pain), metabolic abnormalities (hyperuricemia, hypocalcemia, hypomagnesemia, decreased amino acid levels, acidosis), renal calculi, as well as cardiac abnormalities (cardiomyopathy and prolonged QT interval) [137].

Neuromodulation therapies such as responsive neurostimulation and VNS provide alternatives when surgery is not feasible or has failed, though these methods also have limitations regarding optimal settings and side effects [126,130].

Emerging treatments and complementary therapies, including acupuncture, cannabinoids, and yoga, have been explored for their potential benefits in managing epilepsy. However, these therapies lack robust evidence supporting their efficacy and are often not considered standard treatment options [126]. The need for solid evidence and well-defined mechanisms for these therapies limits their acceptance and use in clinical practice.

Limitations

A major shortcoming of this review is the limited availability of high-quality, RCTs for certain surgical procedures, particularly in the context of long-term outcomes and quality-of-life assessments.

## Conclusions

This review suggests various surgical procedures, including traditional methods of treating epilepsy. Although epilepsy does respond to medication, in refractory cases, numerous surgical methods are being suggested. The clinical implications of surgical procedures, including both invasive and non-invasive methods have been studied and have improved treatment responses. While these procedures have shown promising results in reducing seizure frequency and improving quality of life, several barriers limit their broader application. These include delayed referrals, inconsistent definitions of medically refractory epilepsy, and disparities in access to specialized care. Moreover, the long-term psychosocial and cognitive outcomes of these surgeries remain underexplored, necessitating further research.
